# Fast Genomic Predictions via Bayesian G-BLUP and Multilocus Models of Threshold Traits Including Censored Gaussian Data

**DOI:** 10.1534/g3.113.007096

**Published:** 2013-09-01

**Authors:** Hanni P. Kärkkäinen, Mikko J. Sillanpää

**Affiliations:** * Department of Agricultural Sciences, University of Helsinki, Helsinki FIN-00014; † Department of Mathematical Sciences, University of Oulu, Oulu FIN-90014, Finland; ‡ Department of Biology and Biocenter Oulu, University of Oulu, Oulu FIN-90014, Finland

**Keywords:** genomic selection, multiocus association model, G-BLUP, threshold model, ordinal, binary, censored Gaussian, GenPred, shared data resources

## Abstract

Because of the increased availability of genome-wide sets of molecular markers along with reduced cost of genotyping large samples of individuals, genomic estimated breeding values have become an essential resource in plant and animal breeding. Bayesian methods for breeding value estimation have proven to be accurate and efficient; however, the ever-increasing data sets are placing heavy demands on the parameter estimation algorithms. Although a commendable number of fast estimation algorithms are available for Bayesian models of continuous Gaussian traits, there is a shortage for corresponding models of discrete or censored phenotypes. In this work, we consider a threshold approach of binary, ordinal, and censored Gaussian observations for Bayesian multilocus association models and Bayesian genomic best linear unbiased prediction and present a high-speed generalized expectation maximization algorithm for parameter estimation under these models. We demonstrate our method with simulated and real data. Our example analyses suggest that the use of the extra information present in an ordered categorical or censored Gaussian data set, instead of dichotomizing the data into case-control observations, increases the accuracy of genomic breeding values predicted by Bayesian multilocus association models or by Bayesian genomic best linear unbiased prediction. Furthermore, the example analyses indicate that the correct threshold model is more accurate than the directly used Gaussian model with a censored Gaussian data, while with a binary or an ordinal data the superiority of the threshold model could not be confirmed.

Genomic estimated breeding values based on genome-wide sets of molecular markers have become an essential resource in plant and animal breeding (Eggen 2012; Nakaya and Isobe 2012). The most commonly used approach to predict the genomic breeding values is the genomic best linear unbiased prediction (G-BLUP), a direct descendant of the pedigree-based BLUP model. G-BLUP uses the marker information in estimating realized relationships between the individuals, and then uses the marker-estimated genomic relationship matrix in a mixed model context (*e.g.*, VanRaden 2008; Powell *et al.* 2010). A relatively recent contender for the BLUP-type of model in the genomic selection field is to apply simultaneous estimation and variable selection or variable regularization to multilocus association models (*e.g.*, Meuwissen *et al.* 2001; Xu 2003). Contrary to G-BLUP, a multilocus association model uses the marker information directly by assigning different, possibly zero, effects to the marker genotypes. The genomic breeding value of an individual is then quantified as a sum of the marker effects. Because the number of genetic markers is usually orders of magnitude greater than the number of individuals available for the study, the selection or regularization of the predictors is the key factor of a multilocus association model. In Bayesian genomic selection models the regularization of the excess predictors is performed by shrinking the effects of the markers not linked to the phenotype toward zero by assigning a suitable shrinkage inducing prior density for the marker effects. The most widely used shrinkage inducing priors comprise the Student’s *t* and the Laplace densities, the former of which has been used by Meuwissen *et al.* (2001), by Xu (2003), Yi and Banerjee (2009), Hayashi and Iwata (2010), and Habier *et al.* (2011), whereas the latter has been used, *e.g.*, by de los Campos *et al.* (2009), Meuwissen *et al.* (2009), and Sun *et al.* (2010). The models relying on the Laplace density are commonly denoted as Bayesian LASSO (Park and Casella 2008).

In the basic form of these linear models, the response is assumed continuous with normally distributed residual variation. However, in many instances the actual phenotypic records are given as binary case-control, ordered categorical (*e.g.*, from nonaffected via different severity levels to strongly affected) or censored Gaussian records (*e.g.*, a logarithm of an event history or survival data, or spiked phenotypes as in Broman 2003). With a binary response, either logit or probit transformation is used to convert the binary response into the probability of the positive outcome. Both logit and probit models are developed by assuming an underlying continuous response, often called the latent variable or liability, dichotomized by setting a threshold to limit the two classes. The difference between the logit and probit models is the assumed distribution of the underlying response; the logit model assumes a logistic and the probit model a Gaussian density for the underlying variable. In the frequentist framework, the discrete response is usually modeled without considering the underlying continuous variable, leading to quite different estimation procedure for the model parameters than in linear Gaussian regression. Although under a linear Gaussian model the maximum-likelihood estimate has a closed-form solution, under the logit and probit models the partial derivatives of the likelihood function with respect to the regression coefficients either do not exist or cannot be determined analytically, and the maximum-likelihood estimate must be computed iteratively. The logistic link function leads to somewhat simpler algebraic expressions when handled as an integrated part of the linear model and is therefore often preferred by the frequentists (McCullagh and Nelder 1989).

Contrary to majority of the frequentist models, in the Bayesian context the underlying continuous response is included into the model as a separate latent variable layer, usually assumed to follow a Gaussian density. These two factors, that the augmentation of the latent variable is now an additional layer in the hierarchical model and that the augmented variable is assumed Gaussian, permit the usage of the original linear Gaussian model as such without further modifications. Moreover, in genetics, the normal assumption is especially reasonable as the inheritance of complex traits is determined by multiple genes and environmental factors resulting the liability likely to be normally distributed.

The ordered categorical records can be dichotomized and analyzed with a binary model, or alternatively incorporated as Gaussian observations in a general linear model (*e.g.*, Meijering and Gianola 1985; Wang *et al.* 2013). The problem with the former procedure is that it loses the information contained by the extra categories, whereas in the latter method the model is in effect not compatible with the data. Similarly to a binary phenotype, the ordinal phenotypes can be assumed to have an underlying continuous response discretized by introducing thresholds delimiting the categories (Hackett and Weller 1995). Now there are several thresholds at unknown positions, but otherwise the binary model can be seen as a special case of the ordinal model. The threshold idea can be extended to censored data sets by simply using the uncensored data as such while considering the censored phenotypes as latent variables. The advantage of this approach is that it does not matter which part of the data are censored (right, left, interval, two way censoring), the latent variable is always handled similarly.

Threshold models for ordinal and binary traits have been considered previously by several authors. A threshold model for BLUP with fixed thresholds has been covered by Gianola (1982) and with unknown, estimated threshold positions by Sorensen *et al.* (1995). Multilocus association models of binary and ordinal traits have been considered by Hoti and Sillanpää (2006), Iwata *et al.* (2009), González-Recio *et al.* (2009), González-Recio and Forni (2011), and Wang *et al.* (2013). Furthermore, the threshold approach for censored observations has been considered by Broman (2003), within BLUP context by Sorensen *et al.* (1998), and with multilocus association models by Sillanpää and Hoti (2007) and Iwata *et al.* (2009).

In this article we aim to enlarge on the threshold model more generally. We consider two Bayesian hierarchical models representing the alternative modeling approaches, a Bayesian version of the G-BLUP and a hierarchical Bayesian LASSO (*e.g.*, Park and Casella 2008; Kärkkäinen and Sillanpää 2012a) as a representative of a multilocus association model with variable regularization, and show that in case of a binary, ordinal, or censored Gaussian phenotype the same additional latent variable layer can be plugged into both types of the genomic selection models. In fact, the additional latent variable layer can be subsumed into legions of different linear Gaussian models; Wang *et al.* (2013) have used it with BayesA, BayesB, and BayesC*π*, whereas in our previous work (Kärkkäinen and Sillanpää 2012a) we incorporated a binary threshold-based latent layer into 13 distinct models, including a Bayesian G-BLUP, BayesA, BayesB and both hierarchical and nonhierarchical Bayesian LASSO. In this work, we exemplify the threshold method with a hierarchical Bayesian LASSO as it proved the best working model in the aforementioned previous work and, on the other hand, we did not want to pick anything lesser known, such as the extended Bayesian LASSO (introduced by Mutshinda and Sillanpää 2010, used successfully, *e.g.*, in Kärkkäinen and Sillanpää 2012b) to serve as an example.

A more immediate practical offering of this paper is the fast *maximum a posteriori* (MAP) estimation algorithm presented. Traditionally the parameter estimation for Bayesian models has been performed by finding the posterior density by Markov chain Monte Carlo (MCMC) sampling; however, because of the ever-increasing number of markers available, the focus within the genomic breeding value prediction field has been shifting to the fast MAP estimation methods (*e.g.*, Meuwissen *et al.* 2009; Yi and Banerjee 2009; Hayashi and Iwata 2010; Shepherd *et al.* 2010; Xu 2010; Sun *et al.* 2012). The MAP estimation is based on finding the maximum of the posterior density rather than the whole posterior distribution, usually by an expectation-maximization (EM) algorithm (Dempster *et al.* 1977; McLachlan and Krishnan 1997). The difference in speed between an MCMC and a MAP estimation algorithm is far from trivial: while the run time of an MCMC algorithm is typically hours at the lowest, our generalized expectation maximization (GEM) algorithms perform the example analyses in some 20 sec. With existing genome-wide data sets a fast estimation algorithm is an invaluable asset because it will significantly facilitate the frequent re-estimation of the marker effects and breeding values, the use of cross-validation and permutation-based techniques, and massive simulation studies of breeding programs. Nonetheless, in all of the Bayesian methods for threshold traits found in the literature the parameter estimation has been performed with MCMC. In this respect the methods for discrete data are a bit out of date compared to the methods for Gaussian traits.

In our previous work (Kärkkäinen and Sillanpää 2012a) we already have considered a kindred threshold approach for binary traits in MAP-estimation context. However, an ordinal data set poses an additional challenge because the model has to address the unknown thresholds as well as the latent response. Hence, although a binary phenotype can be regarded as a special case of the ordinal model considered here, a binary model is not readily expandable to several categories. Since the MAP-estimation methods are able to handle large data sets far more efficiently than MCMC methods, it is clear that an applicable MAP-algorithm is needed for all conceivable types of phenotypic observations.

## Materials and Methods

**Figure 1:**
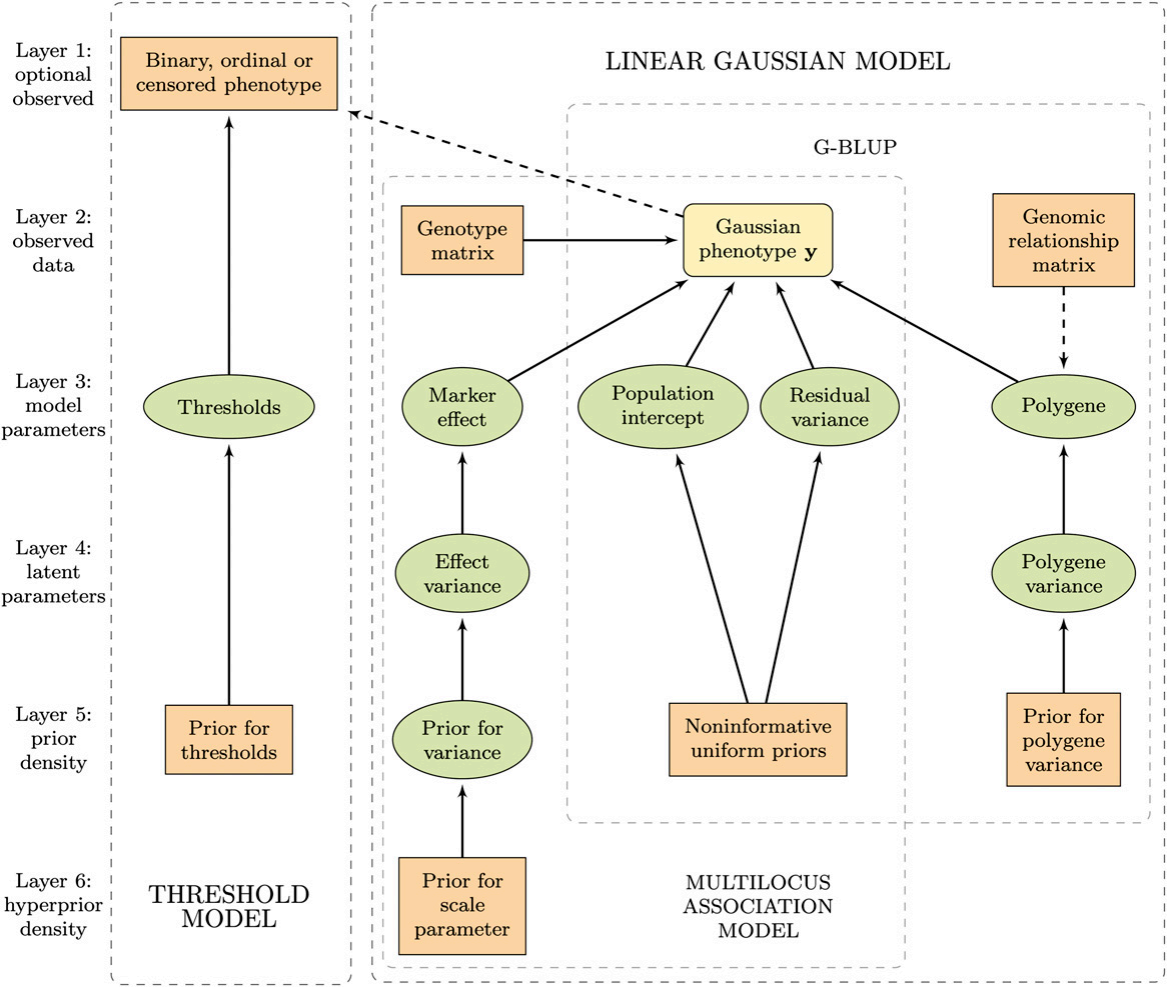
Hierarchical structure of the model framework. The ellipses indicate random parameters and rectangles fixed values, whereas the round-cornered rectangle representing the Gaussian phenotype may be either, depending on whether the threshold module is included in the model. Solid arrows indicate statistical dependency and dashed arrows functional relationship. The background boxes indicate the main modules of the model framework.

Our hierarchical Bayesian model, depicted as a directed acyclic graph in Figure 1, consists of two separate parts, the linear Gaussian model and the threshold model. Under the linear Gaussian model the phenotype measurements are assumed to be continuous and follow a Gaussian density, while the additional threshold model handles binary, ordinal and censored Gaussian observations.

Under the threshold model, we assume that the observed phenotype **w** consists of either ordered categorical or censored Gaussian observations, and that the ordered categorical variable has arisen as an underlying normally distributed continuous response **y** is rendered discrete with a known number of thresholds at unknown positions. The underlying Gaussian response **y** can be explained by genetic factors with either a multilocus association model

$$y=\beta_{0}+X\beta +\varepsilon ,$$ (1)

or with a G-BLUP model

$$y=\beta_{0}+Zu+\varepsilon$$ (2)

(the linear Gaussian model module in Figure 1). Both (1) and (2) are linear Gaussian models commonly used in genomic selection. In the former model (1), the matrix **X** denotes the genotypic records of *p* biallelic single-nucleotide polymorphisms (SNP) of *n* learning set individuals, coded with respect to the number of the rare alleles and standardized to have zero mean and unity variance, and **β** denotes the marker effects. In the latter model (2) the *N*-vector **u** denotes the additive genetic values of the *N* learning and prediction set individuals, whereas **Z** is a *n* × *N* design matrix connecting the genetic values of the *n* learning set individuals to the latent response. Although the additive genetic values of both learning and prediction set individuals are present in the model (2), the latter do not contribute to the likelihood. *β*_0_ denotes the population intercept in both equations. The residuals **ε** are considered independent and identically distributed under both models, with $\varepsilon ∼\text{MVN}\left(0,\sigma_{0}^{2}I_{n}\right)$. To avoid overparametrization and ensure identifiability, the residual variance component $\left(\sigma_{0}^{2}\right)$ is set to unity when the Gaussian response is unobservable (see *e.g.*, Cox and Snell 1989). When the actual Gaussian response is fully observed, the threshold module is omitted from the model (Figure 1) and the residual variance is estimated simultaneously to other model parameters.

In this work the regularization of the excess predictors is performed by a hierarchical Bayesian LASSO (Park and Casella 2008), by specifying a Laplace prior density for the regression coefficients. The Laplace density works best and provides an easy derivation of the fully conditional posterior densities for the effect variances (even though not actual conjugacy) when expressed hierarchically as a scale mixture of normal densities (Park and Casella 2008; de los Campos *et al.* 2009; Kärkkäinen and Sillanpää 2012a). The hierarchical formulation of the prior densities, also known as model or parameter expansion, is a well-known method to simplify MCMC algorithms by transforming the prior into a conjugate and hence enabling Gibbs sampling, and to accelerate convergence of the sampler by adding more working parts and therefore more space for the random walk to move (see *e.g.*, Gilks *et al.* 1996; Gelman *et al.* 2004; Gelman 2004). In our previous work (Kärkkäinen and Sillanpää 2012a), we showed that the hierarchical formulation of the Laplace density is superior to its nonhierarchical counterpart also in EM context. The hierarchy is acquired by setting a Gaussian prior for the marker effects $\beta_{j}|\sigma_{j}^{2}∼\text{N}\left(0,\sigma_{j}^{2}\right)$ and an exponential prior to the effect variances $\sigma_{j}^{2}|\lambda ∼\text{Exp}\left(\lambda^{2}/2\right)$. Unconditionally for the effects this leads to a Laplace density. In Figure 1 the hierarchical formulation is observable as the fourth, latent parameters, layer. The scale parameter *λ*^2^ of the Laplace prior determines the amount of shrinkage introduced by the prior, and hence the sparseness of the model. Because the optimal amount of shrinkage depends on the heritability and the genetic architecture of the trait, the number of markers and amount of linkage disequilibrium (LD) present in the data, the selection of the hyperparameter *λ*^2^ is of central importance. Although some authors prefer to give a fixed value to *λ*^2^ (*e.g.*, Figueiredo 2003; Meuwissen *et al.* 2009; Xu 2010), the most prevalent solution is to estimate it simultaneously to the model parameters (*e.g.*, Yi and Xu 2008; de los Campos *et al.* 2009; Shepherd *et al.* 2010). To this end we give the hyperparameter *λ*^2^ a Gamma(*κ*, *ξ*) hyperprior, and tune the rate parameter *ξ* of the gamma density to a suitable data specific value (sixth layer in Figure 1). The prior densities for the population intercept *β*_0_ and the residual variance $\sigma_{0}^{2}$ (when applicable, *i.e.*, under the Gaussian phenotype model) are uniform *p*(*β*_0_) ∝ 1 and Jeffreys’ $p\left(\sigma_{0}^{2}\right)\propto 1/\sigma_{0}^{2}$, respectively. As the model parameters are considered *a priori* independent, the joint posterior density of the parameter vector becomes
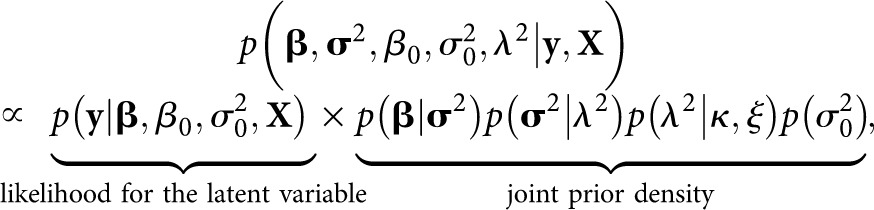
(3)where $\sigma^{2}=\left(\sigma_{1}^{2},\ldots ,\sigma_{p}^{2}\right)$ is a vector of the marker variances.

Under the Bayesian G-BLUP (2) the prior density for the genetic values **u** is a conjugate multivariate normal $\text{MVN}\left(0,G\sigma_{u}^{2}\right)$, where the realized relationship matrix **G** has been estimated from the marker data. In this work the estimation has been performed with the second method described in VanRaden (2008). Contrary to the classical framework, in a Bayesian approach the variance components are estimated simultaneously with the genomic breeding values (Hallander *et al.* 2010; Kärkkäinen and Sillanpää 2012a). The genetic variance component $\sigma_{u}^{2}$ has been given a flat Inverse-*χ*^2^(*ν*, *τ*^2^) prior distribution with a substantially large *τ*^2^ to ensure the flatness of the density. Similarly to the multilocus association model, the prior densities for the population intercept and the residual variance (under the Gaussian phenotype model) are *p*(*β*_0_) ∝ 1 and $p\left(\sigma_{0}^{2}\right)\propto 1/\sigma_{0}^{2}$. The joint posterior density of the G-BLUP model parameters is given by


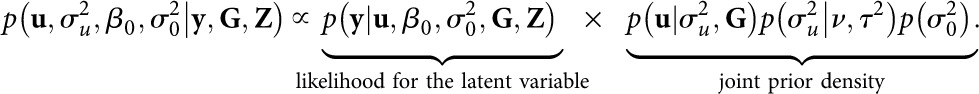
(4)

Given the value of the continuous, normally distributed latent variable *y_i_*, the binary or ordinal variable *w_i_* has value *k* ∈ {1, …, *K*} with a probability

$$\text{P}\left(w_{i}=k|y_{i},t_{k-1},t_{k}\right)=\{\begin{array}{ll} 1, & \text{when}\;t_{k-1}<y_{i}<t_{k}\\ 0, & \text{otherwise}, \end{array}$$ (5)

where *t_k_*_−1_ and *t_k_* are the thresholds delimiting the *k*th category. If the ordinal variable has *K* categories, there will be *K* + 1 thresholds, such that **t** = {(*t*_0_, *t*_1_, …, *t_K_*)|*t*_0_ < *t*_1_ < … < *t_K_*, *t*_0_ = −∞, *t*_1_ = 0, *t_K_* = ∞}. One of the thresholds must be fixed in order to center the underlying distribution; we adopt the common fashion to set *t*_1_ into zero (*e.g.*, Cox and Snell 1989; Sorensen *et al.* 1995). The *K* − 2 of the thresholds **t**^⋆^ = {(*t*_2_, …, *t_K_*_−1_)|*t*_2_ < … < *t_K_*_−1_} are considered unknown, and are estimated simultaneously to the model parameters. With a binary response (*K* = 2) there obviously are no unknown threshold values.

Although the likelihood of the observed phenotype **w** follows a categorical density, conditionally on the underlying response and the thresholds the observed ordinal phenotype is known with certainty and hence the likelihood P(*w_i_* = *k*|*y_i_*, **t**) gets a constant value, zero or one. Therefore, the fully conditional posterior density of the latent Gaussian variable *y_i_*, given the value of the ordinal observation *w_i_*, corresponds the prior density of *y_i_* when *t_k_*_−1_ < *y_i_* < *t_k_* and is zero otherwise. As the latent variable is an additional layer in the hierarchical model, the prior density for the underlying Gaussian response **y** is the likelihood of the latent variable under the linear Gaussian models (1) or (2). The likelihood of the latent Gaussian variable, given by the model equations (1) or (2) and the assumptions below them, is a multivariate normal centered at *β*_0_ + **Xβ** under the multilocus association model and at *β*_0_ + **Zu** under the G-BLUP model, respectively, the covariance being set to unity under both models. Hence, the fully conditional posterior density of *y_i_* is a truncated normal distribution (truncated at points *t_k_*_−1_ and *t_k_*) with a density function (for simplicity, the ⋆ denotes the data and all other parameters)

$$p\left(y_{i}|⋆\right)=\frac{\phi \left(y_{i}-E\left(y_{i}\right)\right)}{\Phi \left(t_{k}-E\left(y_{i}\right)\right)-\Phi \left(t_{k-1}-E\left(y_{i}\right)\right)},$$ (6)

where *ϕ*(⋅) and Φ(⋅) denote the standard normal density and cumulative distribution functions, respectively, while E(*y_i_*) is the linear predictor of the model (1) or (2).

Following Sorensen *et al.* (1995) the prior for the *K* − 2 unknown thresholds **t**^⋆^ = (*t*_2_, …, *t_K_*_−1_) has been given as order statistics from an Uniform(0, *t_max_*) distribution,

$$p\left(t^{⋆}|⋆\right)=\left(K-2\right)!{\left(\frac{1}{t_{max}}\right)}^{K-2}\text{for}\;0<t_{2}<\ldots t_{K-1}<t_{max},\;\text{and}\;0\;\text{otherwise}.$$ (7)

The fully conditional posterior density for a *t_k_* is proportional to the product of the prior and the likelihood of the observed ordinal phenotype **w**. Note, that the threshold values **t**^⋆^ appear in the prior density (7) only at the definition of the support of the distribution. As the terms not including the parameter are discarded as constants from the fully conditional posterior, the support definition is all that passes from the prior to the posterior. Therefore, the fully conditional posterior density for a *t_k_* is given by the likelihood of the observed ordinal phenotype **w**, within the set of values determined by the prior density of **t**^⋆^,

$$\begin{array}{c} p\left(t_{k}|⋆\right)\propto p\left(w|y,t\right)p\left(t⋆\right)\propto \prod_{i=1}^{n}\text{P}{\left(w_{i}=k\right)}^{\text{I}\left(w_{i}=k\right)}\text{P}{\left(w_{i}=k+1\right)}^{\text{I}\left(w_{i}=k+1\right)}\\ =\prod_{i=1}^{n}\text{P}{\left(t_{k-1}<y_{i}<t_{k}|t_{k-1},t_{k}\right)}^{\text{I}\left(w_{i}=k\right)}\text{P}{\left(t_{k}<y_{i}<t_{k+1}|t_{k},t_{k+1}\right)}^{\text{I}\left(w_{i}=k+1\right)} \end{array}$$ (8)

for 0 < *t*_2_ < … *t_K_*_−1_ < *t_max_* and 0 otherwise. If (8) is seen as a function of *t_k_*, it can be seen that the value of *t_k_* must be larger that all of the *y_i_*|*w_i_* = *k* and smaller than all of the *y_i_*|*w_i_* = *k* + 1. Hence, as a function of *t_k_*, (8) leads to the uniform density

$$p\left(t_{k}|⋆\right)=\frac{1}{\text{min}\left(y_{i}|w_{i}=k+1\right)-\text{max}\left(y_{i}|w_{i}=k\right)}.$$ (9)

For a Gaussian phenotype with censored observations we define an additional binary variable *ω_i_* = 1 if the *i*th observation is censored and *ω_i_* = 0 if not. As the threshold model assumes an unity variance for the latent Gaussian response, the observed phenotype must be standardized accordingly. This is done by regarding the available observations as a sample from a truncated normal density and using the connection between the quantiles and the standard deviation of a Gaussian density (*e.g.*, 25% of the observations are ≤ *μ* − 0.67*σ*, or 15.73% are ≤ *μ* − *σ*). Now, if *ω_i_* = 0 the standardized Gaussian phenotype is used directly, and if *ω_i_* = 1 the underlying uncensored response is computed as previously. The latent variable parametrization of the binary phenotype is similar to a generalized linear model with the probit link function (Albert and Chib 1993), whereas the parametrization of the censored phenotype corresponds to a generalized linear model with the tobit link function (see *e.g.*, Tobin 1958; Sorensen *et al.* 1998; Iwata *et al.* 2009).

The model parameters are estimated by the GEM (Neal and Hinton 1999) algorithm, which is presented in the Appendix A2. The algorithm finds a *maximum a posteriori* point estimate for each of the parameters and latent variables by repeatedly updating the parameters one at the time to their conditional expectations (see Kärkkäinen and Sillanpää 2012a). Due to the conjugate or otherwise suitable prior densities chosen, the fully conditional posterior densities for the parameters and the latent Gaussian response are known probability density functions. This guarantees an easy derivation of the GEM-algorithm; as the expected values of the known densities are automatically available, we do not need to find the fully conditional posterior expectations by integration. In addition, if preferred it would be trivial to implement an MCMC Gibbs sampler to sample from these densities. The fully conditional posterior densities for the latent Gaussian response and for the unknown thresholds are given in the aforementioned models (6) and (9), whereas the fully conditional posterior densities for the parameters of the linear Gaussian models can be easily extracted from the joint posterior densities of the models (3) and (4). The derivations of the fully conditional posterior densities of the multilocus association model are presented in the Appendix A1.

The fully conditional posterior densities for the multilocus association model (1) parameters are as follows. The fully conditional posterior density for a marker effect *β_j_* is normal

$$\begin{array}{c} \beta_{j}|⋆∼\text{N}\left(\mu_{j},s_{j}^{2}\right),\;\text{with}\\ \mu_{j}=\sum_{i=1}^{n}x_{ij}\left(y_{i}-\beta_{0}-\sum_{l\neq j}\beta_{l}x_{il}\right)/\left(\sum_{i=1}^{n}{\left(x_{ij}\right)}^{2}+\frac{\sigma_{0}^{2}}{\sigma_{j}^{2}}\right),\\ s_{j}^{2}=\sigma_{0}^{2}/\left(\sum_{i=1}^{n}{\left(x_{ij}\right)}^{2}+\frac{\sigma_{0}^{2}}{\sigma_{j}^{2}}\right), \end{array}$$ (10)

where the residual variance $\sigma_{0}^{2}=1$ unless the actual Gaussian phenotype is observed. The fully conditional posterior density for the inverse of a marker variance is an inverse-Gaussian (Chhikara and Folks 1989)

$$\frac{1}{\sigma_{j}^{2}}|⋆∼\text{Inverse-Gaussian}\left(\frac{\lambda }{\left|\beta_{j}\right|},\lambda^{2}\right).$$ (11)

The fully conditional posterior density for the LASSO parameter *λ*^2^ is a gamma density

$$\lambda^{2}|⋆∼\text{Gamma}\left(\kappa +p,\xi +\sum_{j=1}^{p}\frac{\sigma_{j}^{2}}{2}\right).$$ (12)

The fully conditional posterior density for the population intercept equals a normal density

$$\beta_{0}|⋆∼\text{N}\left(\frac{1}{n}\sum_{i=1}^{n}\left(y_{i}-\sum_{j=1}^{p}x_{ij}\beta_{j}\right),\frac{\sigma_{0}^{2}}{n}\right),$$ (13)

where again the residual variance $\sigma_{0}^{2}=1$ unless the actual Gaussian phenotype is observed. Finally, when estimated, the fully conditional posterior density of the residual variance is an inverse-*χ*^2^

$$\sigma_{0}^{2}|⋆∼\text{Inv-}\chi^{2}\left(n,\frac{1}{n}\sum_{i=1}^{n}{\left(y_{i}-\beta_{0}-\sum_{j=1}^{p}x_{ij}\beta_{j}\right)}^{2}\right).$$ (14)

The fully conditional posterior densities for the Bayesian G-BLUP (2) parameters are the following. The fully conditional posterior density for the additive genetic values is a multivariate normal

$$\begin{array}{c} u|⋆∼\text{MVN}\left(\mu_{u},\Sigma_{u}\right),\text{where}\\ \mu_{u}={\left({\mathrm{Z\prime}}^{\prime }Z+\frac{\sigma_{0}^{2}}{\sigma_{u}^{2}}G^{-1}\right)}^{-1}{\mathrm{Z\prime}}^{\prime }\left(y-\beta_{0}\right)\\ \Sigma_{u}={\left(\frac{1}{\sigma_{0}^{2}}{\mathrm{Z\prime}}^{\prime }Z+\frac{1}{\sigma_{u}^{2}}G^{-1}\right)}^{-1}, \end{array}$$ (15)

where the residual variance $\sigma_{0}^{2}$ is again one if the actual Gaussian phenotype is not observed. The fully conditional posterior density for the additive genetic variance is an inverse-*χ*^2^ density

$$\sigma_{u}^{2}|⋆∼\text{Inv-}\chi^{2}\left(\nu +N,\frac{{\mathrm{u\prime}}^{\prime }G^{-1}u+\nu \tau^{2}}{\nu +N}\right),$$ (16)

where the capital *N* denotes the total number of individuals in the learning and test sets. The fully conditional posterior density for the population intercept is normal

$$\beta_{0}|⋆∼\text{N}\left(\frac{1}{n}\sum_{i=1}^{n}\left(y_{i}-u_{i}\right),\frac{\sigma_{0}^{2}}{n}\right),$$ (17)

where the residual variance $\sigma_{0}^{2}=1$ unless the actual Gaussian phenotype is observed. When estimated, the fully conditional posterior density of the residual variance is an inverse-*χ*^2^

$$\sigma_{0}^{2}|⋆∼\text{Inv-}\chi^{2}\left(n,\frac{1}{n}{\left(y-\beta_{0}-Zu\right)}^{\prime }\left(y-\beta_{0}-Zu\right)\right).$$ (18)

The generalized expectation maximization algorithms presented in the Appendix A2 work by updating the parameters to the expected values of the aforementioned fully conditional posterior densities. In our method, as in nearly all Bayesian approaches, the user has to provide some, usually data specific, parameter values for the hyperprior densities at the very bottom of the model hierarchy. In the multilocus association model the hyperprior parameters for the LASSO parameter *λ*^2^ ∼ Gamma(*κ*, *ξ*) have to be given by the user, whereas in the Bayesian G-BLUP this role falls to the hyperprior parameters of the additive genetic variance component $\sigma_{u}^{2}∼\text{Inv-}\chi^{2}\left(\nu ,\tau^{2}\right)$. The selection of the data specific hyperprior parameters is called tuning of the algorithm. The tuning is the easier to perform the fewer parameters there are to be tuned. Because the number of markers (*p*) is very large, the impact of *κ* into the fully conditional posterior expectation of the LASSO parameter $E\left(\lambda^{2}|⋆\right)=\left(\kappa +p\right)/\left(\xi +\sum \sigma_{j}^{2}/2\right)$, derived from the fully conditional posterior density in (12), is obviously negligible. As the only information the GEM algorithm uses in the update process is the fully conditional expectation, we shall simplify the tuning by setting the value of *κ* to a constant value *κ* = 1. Thereby, the rate parameter *ξ* is the only entity in the model to which the user has to provide a data specific value. Accordingly, under the Bayesian G-BLUP the degrees of freedom *ν* of the inverse-*χ*^2^ density do not have a substantial contribution to the fully conditional posterior expectation of the additional genetic variance $E\left(\sigma_{u}^{2}|⋆\right)=\left(u^{\prime }G^{-1}u+\nu \tau^{2}\right)/\left(\nu +N-2\right)$, and we therefore set permanently *ν* = 2, while the scale parameter *τ*^2^ may need data specific tuning.

## Example analyses

In our example analyses, we have considered the predictive performance of the Bayesian multilocus association model and the Bayesian G-BLUP *per se* and with the three different latent variable layers, with two different data sets.

The first of the data sets consists of a simulated data introduced in the XII QTL-MAS Workshop 2008 (Lund *et al.* 2009). The data set can be downloaded from the workshop homepage at http://www.computationalgenetics.se/QTLMAS08/QTLMAS/DATA.html. There are 5865 individuals from seven generations of half sib families with information on 6000 biallelic SNP loci, the loci are evenly distributed over six chromosomes of length 100 cM each (see Lund *et al.* 2009 for details). Since SNPs with minor allele frequency <0.05 within the learning set were discarded, the actual number of markers in the analysis is 5726. The first four generations of the data, 4665 individuals, have both marker information and a phenotypic record, and function as a learning set, whereas the generations five to seven, comprising 1200 individuals, are treated as a prediction set. There are 48 simulated quantitative trait loci (QTL) in the data set, with allele substitution effects drawn from a Gamma(0.42, 1.85) distribution (with shape and rate parametrization). The cumulative effect of the simulated QTL equals the genetic value of the individuals, while the phenotypes of the individuals have been obtained as the sum of the individuals’ genetic value and a random residual drawn from a normal distribution with mean zero and a variance set to produce heritability value 0.3 (Lund *et al.* 2009). As in our previous works, we have generated 100 replicates of the data set by resampling the residuals from a normal density N(0, *var*(*TBV*)(1/*h*^2^ − 1)), where *var*(*TBV*) denotes the observed variance of the genetic values and the heritability *h*^2^ equals 0.3 (Kärkkäinen and Sillanpää 2012a,b). After this each of the generated phenotype sets was scaled to have zero mean and unity variance. The advantage of using a simulated data set in the example analysis is the availability of the true genetic values of the individuals, enabling us to determine the accuracy of the estimates by a direct comparison of the simulated and estimated genetic values.

The second data set, described in detail by Cleveland *et al.* (2012), is a real pig (*Sus scrofa*) data, provided by the Genetics Society of America to be used for benchmarking of genomic selection methods. The pig dataset consists of phenotypic records of 3184 individuals for a quantitative trait (standardized to zero mean and unity variance) with predetermined heritability 0.62, and genotypic records for 60k biallelic SNP markers (45,317 with minimum allele frequency over 0.05 actually included in the analysis). Contrary to the simulated data set, there are neither true genetic values of the individuals nor true effects of the QTL available, and hence we estimate the accuracy of the predicted genomic breeding values by dividing the correlation between the estimates and the original Gaussian phenotypic values by the square root of the predetermined heritability of the trait (Legarra *et al.* 2008). Since the data does not consist a separate validation population we compute the result statistics using cross-validation, where the 3184 individuals are randomly partitioned into 10 subsets (10-fold cross-validation) of 318 or 319 individuals. At each round 9 of the sets are treated as a learning set and the remaining one as the prediction set.

The binary, ordinal, and censored phenotypes of the data sets were constructed as follows. We tested two binary phenotypes with success probabilities 50% and 80%, two ordinal phenotypes with four classes, and three right censored phenotypes consisting of 20%, 50%, or 80% of censored observations. The proportions of observations belonging into each class of the four-class phenotype were either even 20:30:30:20% of observations in each class, or highly unbalanced with 70% belonging to the first class and 10% in the subsequent three classes. The value of the censored observations was set to equal the largest of the noncensored values, leading to a spiked Gaussian phenotype (see Broman 2003). The binary and the evenly distributed ordinal data sets are generated in preparation for an easy ascertainment of the extra power acquired by using the category information compared to the dichotomized phenotype. The binary phenotype with 80% success probability simply sets the first category of the ordinal phenotype as a failure and the subsequent three classes as a success, while the binary response with 50% success probability sets the first and second category as a failure and the third and fourth as a success. The same holds true for the censored data, as the threshold values are set to correspond the thresholds of the binary phenotype: the 20% and 80% censored data can be compared with the binary data with 80% success rate, as the proportions of the observations belonging to the classes is 20:80, and similarly the 50% censored data are equivalent to the 50% or 50:50 binary data. All threshold values were determined as standard normal distribution function parameters leading to the desired threshold value, *e.g.*, a threshold at 0.84, leading to 20% success probability, since Φ(0.84) = 0.8.

The multilocus models are not able to handle an unlimited number of loci with respect to the sample size. Hoti and Sillanpää (2006) have proposed an upper limit of 10 times more loci than individuals, but it seems that in practice a smaller number of loci might be optimal (*e.g.*, real data analysis in Kärkkäinen and Sillanpää 2012a). Furthermore, to our experience, the best results are necessarily not acquired by using as many markers as the model can possible handle, but with a significantly smaller marker set (results not shown). In the QTL-MAS data, the proportion of markers to individuals is almost one-to-one, and no extra measures are needed, but with the pig data the multilocus association model becomes too oversaturated to function properly. Therefore, with the real data, in the beginning of each cross-validation round the number of SNPs is first reduced from 45,317 to 10,000 by the sure independence screening method (Fan and Lv 2008). The method works by ranking the markers with respect to their marginal correlation with the phenotype within the current learning set, and selecting the 10,000 best ranking markers to the multilocus association model. The marginal correlation is computed as the Pearson’s product-moment coefficient, by using the same phenotypic records (binary, ordinal, censored or Gaussian) as is used in the actual multilocus association model, except in the case of the 80% censored Gaussian phenotype, where it proved better to dichotomize the phenotype by setting the uncensored data into zero. In Kärkkäinen and Sillanpää (2012a) we performed the preselection in advance and used the same set of markers in all cross-validation rounds, whereas here the preselection is integrated into the cross-validation procedure. The correlation produced by the former approach appeared to be slightly overestimated. An advantage of the G-BLUP over the multilocus association model is that no preselection of the markers is needed. Some authors have found out that preselection of the markers might have a positive impact also to G-BLUP (see Resende *et al.* 2012), but we did not observe such a behavior (results not shown).

The hyperprior parameters for the example analyses are selected to produce best accuracy. The tuning is performed by testing different parameter values and choosing the one resulting the best correlation between the estimated and the true genetic values. In practice, we simply select two arbitrary values for the parameter, observe the correlation acquired under these values, and proceed to search for an optimal value to the direction pointed by the better performing one. This step could be automatized, but so far we have performed it manually. As we have used both learning and prediction sets in the parameter tuning, the obtained accuracies must be considered as best-case scenarios. Under the multilocus association model (1), we give the LASSO parameter *λ*^2^ a Gamma(1, *ξ*) hyperprior and tune the rate (*ξ*) of the gamma density into a data specific value, while under the G-BLUP model (2) the scale hyperparameter *τ*^2^ for the genetic variance is tuned. The values selected for *ξ* and *τ*^2^ and some of the proposed values, along with the corresponding accuracy estimates under the multilocus association model and the Bayesian G-BLUP, are given in Tables 1 and 2, respectively.

**Table 1:** Hyperprior selection for the Bayesian LASSO

	Data Type							
	Binary		Ordinal		Censored			
Data/Model	50%	80%	Even	Odd	20%	50%	80%	Gaussian
QTL-MAS								
TH	0.20/0.81	0.20/0.75	0.20/0.86	0.20/0.82	0.20/0.86	0.20/0.85	0.20/0.81	−
	**0.50/0.85**	0.50/0.81	**0.50/0.88**	**0.50/0.85**	0.50/0.88	**0.50/0.87**	**0.50/0.83**	−
	1.00/0.84	**1.00/0.82**	1.00/0.87	1.00/0.84	**1.00/0.88**	1.00/0.87	1.00/0.81	−
	2.00/0.82	2.00/0.80	2.00/0.85	2.00/0.81	2.00/0.87	2.00/0.86	2.00/0.78	−
G	0.05/0.84	0.02/0.79	0.10/0.85	0.10/0.80	0.05/0.84	0.05/0.85	0.01/0.76	0.10/0.87
	**0.07/0.85**	**0.05/0.82**	0.20/0.87	0.20/0.84	0.10/0.87	**0.10/0.86**	0.02/0.79	0.20/0.88
	0.10/0.84	0.10/0.80	**0.30/0.88**	**0.30/0.84**	**0.25/0.88**	0.25/0.83	**0.03/0.80**	**0.30/0.89**
	0.20/0.81	0.20/0.75	0.60/0.87	0.60/0.82	0.50/0.86	0.50/0.79	0.06/0.77	0.60/0.88
Pig								
TH	1.00/0.51	3.00/0.48	1.00/0.56	1.00/0.55	1.00/0.56	1.00/0.54	1.00/0.47	−
	**3.00/0.55**	**5.00/0.49**	3.00/0.59	**3.00/0.56**	3.00/0.59	**3.00/0.57**	**3.00/0.49**	−
	5.00/0.55	7.00/0.49	**5.00/0.59**	5.00/0.55	**5.00/0.60**	5.00/0.57	5.00/0.48	−
	7.00/0.54	9.00/0.48	7.00/0.58	7.00/0.54	7.00/0.59	7.00/0.56	7.00/0.48	−
G	0.10/0.51	0.10/0.46	0.50/0.56	1.00/0.55	0.20/0.55	0.20/0.53	0.04/0.41	0.50/0.59
	0.20/0.55	**0.15/0.48**	1.00/0.59	1.50/0.56	0.40/0.58	**0.30/0.54**	**0.05/0.42**	**1.00/0.61**
	**0.30/0.55**	0.20/0.47	**1.50/0.59**	**2.00/0.56**	**0.60/0.59**	0.40/0.54	0.06/0.41	1.50/0.59
	0.40/0.53	0.25/0.46	2.00/0.57	2.50/0.56	0.80/0.59	0.50/0.51	0.07/0.41	2.00/0.57

Different values given for the scale parameter *ξ* of the gamma hyperprior for the LASSO parameter, and the corresponding average accuracy of the genomic breeding value estimates (*ξ*/accuracy) within the 100 QTL-MAS data replicates and the 10 cross-validation partitions of the pig data set. The boldface values are the ones selected for the analyses. “Model” refers to the model type used, TH being the correct threshold model and G the linear Gaussian model used directly. The correlation in the pig data is computed as correlation between the estimated genomic breeding values and the Gaussian phenotypes divided by the square root of the predetermined heritability 0.62. The ”Binary” phenotype has either 50% or 80% success probability. The class sizes of the “Ordinal” phenotype are “Even,” 20:30:30:20%, and “Odd,” 70:10:10:10%. The percentage of censored observations in the “Censored” phenotype is 20%, 50%, or 80%. ”Gaussian” refers to the original fully observed Gaussian phenotype

**Table 2:** Prior selection for the Bayesian G-BLUP

	Data Type							
	Binary		Ordinal		Censored			
Data/Model	50%	80%	Even	Odd	20%	50%	80%	Gaussian
QTL-MAS								
TH	400/0.74	400/0.71	400/0.78	400/0.74	400/0.78	400/0.77	400/0.72	−
	800/0.75	800/0.72	**800/0.79**	800/0.75	**800/0.80**	**800/0.78**	**800/0.74**	−
	**1000/0.75**	**1000/0.72**	1000/0.79	**1000/0.75**	1000/0.80	1000/0.78	1000/0.73	−
	1200/0.75	1200/0.72	1200/0.79	1200/0.75	1200/0.79	1200/0.78	1200/0.73	−
G	25/0.70	10/0.64	200/0.77	200/0.73	100/0.76	50/0.74	10/0.67	200/0.78
	50/0.74	25/0.71	400/0.79	**400/0.74**	200/0.79	100/0.77	**25/0.71**	**400/0.80**
	**100/0.75**	**50/0.72**	**600/0.79**	600/0.74	**400/0.79**	**200/0.77**	50/0.70	500/0.80
	200/0.74	100/0.71	800/0.79	800/0.73	600/0.79	400/0.75	100/0.66	600/0.80
Pig								
TH	400/0.56	400/0.54	400/0.60	400/0.59	400/0.61	400/0.60	400/0.55	−
	800/0.58	800/0.55	800/0.62	**800/0.60**	800/0.62	800/0.61	**800/0.55**	−
	**1200/0.58**	**1000/0.55**	**1000/0.62**	1000/0.60	**1000/0.62**	**1000/0.61**	1000/0.55	−
	1600/0.56	1200/0.55	1200/0.62	1200/0.59	1200/0.62	1200/0.61	1200/0.55	−
G	25/0.55	10/0.49	200/0.60	200/0.56	100/0.59	50/0.57	5/0.47	200/0.62
	50/0.57	25/0.54	**400/0.61**	400/0.59	**200/0.61**	**100/0.58**	**10/0.50**	**400/0.63**
	**100/0.57**	**50/0.54**	600/0.60	**600/0.59**	300/0.61	150/0.57	25/0.49	500/0.62
	200/0.34	100/0.51	800/0.41	800/0.59	400/0.49	200/0.49	50/0.44	600/0.44

Different values given for the scale parameter *τ*^2^ of the inverse-*χ*^2^ prior for the polygene variance, and the corresponding average accuracy of the genomic breeding value estimates (*τ*^2^/accuracy) within the 100 QTL-MAS data replicates and the 10 cross-validation partitions of the pig data set. The boldface values are the ones selected for the analyses. The column “**Model**” refers to the model type used, TH being the correct threshold model and G the linear Gaussian model used directly. The correlation in the pig data is computed as correlation between the estimated genomic breeding values and the Gaussian phenotypes, divided by the square root of the predetermined heritability 0.62. The “Binary” phenotype has either 50% or 80% success probability. The class sizes of the “Ordinal” phenotype are “Even,” 20:30:30:20%, and “Odd,” 70:10:10:10%. The percentage of censored observations in the “Censored” phenotype is 20%, 50%, or 80%. ”Gaussian” refers to the original fully observed Gaussian phenotype

Comparing the relative performance of the multilocus association model and the Bayesian G-BLUP with the two data sets (Table 3) clearly shows that the multilocus association model is superior when the trait is controlled by a moderate number of genes (QTL-MAS data), whereas the G-BLUP is a reasonable choice when the trait is either truly polygenic or there is strong linkage disequilibrium present (the pig data). With the 100 Gaussian QTL-MAS data replicates, the multilocus association model produces an average correlation 0.89 whereas the G-BLUP produces an average correlation 0.80. Consistently, with all of the binary, ordinal, and censored Gaussian QTL-MAS data sets, there is an approximately 10-point difference in favor of the multilocus association model in the average correlation. Regarding the pig data, the G-BLUP has the advantage over the multilocus association model: the average correlation in the 10 cross-validation sets is three points higher with the fully observed Gaussian phenotype and two to four points higher with most of the other phenotypes. The advantage of the G-BLUP is even more significant with the 80% success rate binary phenotype and the 80% censored phenotype, the G-BLUP being on average six points more accurate. In this case, however, the culprit is not only the multilocus association model itself but also the sure independence screening used beforehand to reduce the number of markers: if the marginal correlation was computed by using the Gaussian phenotype instead of the binary, the final average correlation would be more consistent 0.51 instead of the now observed 0.49 (data not shown).

**Table 3:** Model accuracy

	Binary		Ordinal		Censored			
Data/Model	50%	80%	Even	Odd	20%	50%	80%	Gaussian
Bayesian LASSO								
QTL-MAS								
TH	0.85 ± 0.02	0.82 ± 0.02	0.88 ± 0.01	0.85 ± 0.02	0.88 ± 0.01	0.87 ± 0.01	0.83 ± 0.02	−
G	0.85 ± 0.02	0.82 ± 0.02	0.88 ± 0.01	0.84 ± 0.02	0.88 ± 0.01	0.86 ± 0.02	0.80 ± 0.03	0.89 ± 0.01
Pig								
TH	0.55 ± 0.03	0.49 ± 0.05	0.59 ± 0.03	0.56 ± 0.04	0.60 ± 0.03	0.57 ± 0.05	0.49 ± 0.04	−
G	0.55 ± 0.03	0.48 ± 0.05	0.59 ± 0.03	0.56 ± 0.04	0.59 ± 0.03	0.54 ± 0.05	0.42 ± 0.05	0.61 ± 0.03
Bayesian G-BLUP								
QTL-MAS								
TH	0.75 ± 0.02	0.72 ± 0.03	0.79 ± 0.02	0.75 ± 0.02	0.80 ± 0.02	0.78 ± 0.02	0.74 ± 0.02	−
G	0.75 ± 0.02	0.72 ± 0.03	0.79 ± 0.02	0.74 ± 0.02	0.79 ± 0.02	0.77 ± 0.02	0.71 ± 0.03	0.80 ± 0.02
Pig								
TH	0.58 ± 0.04	0.55 ± 0.05	0.62 ± 0.04	0.60 ± 0.04	0.62 ± 0.04	0.61 ± 0.04	0.55 ± 0.05	−
G	0.57 ± 0.04	0.54 ± 0.05	0.61 ± 0.04	0.59 ± 0.04	0.61 ± 0.04	0.58 ± 0.04	0.50 ± 0.05	0.63 ± 0.04

Correlation coefficients (± 1 SD) between the true and estimated genomic breeding values in the 100 replicates of the QTL-MAS data set and the 10 cross-validation partitions of the pig data set. “Model” refers to the model type used, TH being the correct threshold model and G the linear Gaussian model used directly. The correlation in the pig data is computed as correlation between the estimated genomic breeding values and the Gaussian phenotypes, divided by the square root of the predetermined heritability 0.62. The “Binary” phenotype has either 50% or 80% success probability. The class sizes of the “Ordinal” phenotype are “Even,” 20:30:30:20% and “Odd,” 70:10:10:10%. The percentage of censored observations in the “Censored” phenotype is 20%, 50%, or 80%. “Gaussian” refers to the original fully observed Gaussian phenotype

As the binary and the evenly distributed ordinal data sets are related to each other it is easy to ascertain the extra power acquired by using the category information compared with the dichotomized phenotype. Table 3 shows a significantly improved accuracy if the additional categories are taken into account: with the ordinal data the mean correlation is 6–10 points or 7–20% higher than with the 80% success rate binary data, and 3–4 points or 5–7% higher than with the 50% success rate binary data. The percentage advantage is greater in situations in which the power of the analysis is lower. As expected, the accuracy is lower with the 70:10:10:10% ordinal phenotype than with the evenly distributed ordinal phenotype, the difference being 2–4 points with both data sets under both models. The additional accuracy gained by using the correct model (*i.e.*, the threshold model) for the binary and ordinal phenotypes, instead of using the linear Gaussian model directly, was minor. The threshold model was a trifle of more accurate in some cases (Table 3). The correlation obtained with the threshold model was one point greater in 7 cases of 16, more often with the unevenly distributed responses (binary 80% and ordinal 70:10:10:10) than with the evenly distributed (binary 50% and ordinal 20:30:30:20); with the unevenly distributed phenotypes there was five cases in which a modicum of extra accuracy was gained with the threshold model, whereas with the evenly distributed there was only two. The extra accuracy was also observed more often with the pig data than with the QTL-MAS data (five and two cases, respectively), and with the Bayesian G-BLUP than with the Bayesian LASSO (also five and two cases, respectively).

The censored data sets consist of a continuous normally distributed phenotype with 20%, 50%, or 80% right censored observations, set to equal the maximum of the non-censored observations. The non-censored observations clearly contain extra information compared to the corresponding binary data (Table 3). The correlations acquired with the data sets with 20% censored observations were in all cases considerably, 6–11 points or 6–22%, higher than with the corresponding 20:80 binary data sets. With the 50% censored data sets the difference is 1–3 points, or 1–5%, compared to the 50:50 binary data. Even the data sets with 80% censored observations may be slightly more informative than the 20:80 binary data, the correlation being one point in favor of the censored phenotype with the QTL-MAS data under the threshold LASSO and threshold G-BLUP. The 20% censorship weakens the accuracy only slightly compared to the fully observed Gaussian phenotype: no more than one point if the threshold model is used, and 1–2 points if the Gaussian model is used directly for the censored phenotype. Contrary to the ordinal-phenotype-case, with a censored phenotype the threshold model is clearly more accurate than the Gaussian model (Table 3). The difference between the models with the data sets with 20% censored phenotypes is tiny (one point in three cases out of four), but increases when the censoring grows stronger. With 50% censored observations the difference is 1–3 points or 1–5%, and with 80% censored observations 3–7 points, or 4–17%.

The steps of the GEM algorithm are repeated until convergence. The algorithm is considered to be converged when the correlation between the estimated breeding values of two consecutive iterations is greater than 1−10^−6^. The convergence is confirmed visually by examining the behavior of parameter values during the iterations and verifying that all of the parameters have reached a constant level; this is also how the suitable value for the convergence rule has been originally ascertained. The required number of iterations is usually between 40 and 80 under the multilocus association model, and around 10 under the G-BLUP. So far we have not encountered problems in the convergence, given that appropriate hyper(prior) parameter values have been selected, and that the number of markers with respect to the sample size in the multilocus association model has not been too large. Depending on the data and the model variate, the computation time is around 15–50 sec with a 64-bit Windows 7 desktop computer with 3.50 GHz Intel(i7) CPU and 16.0 GB RAM.

## Discussion

Our example analyses show that using the extra classes and the uncensored observations present in an ordered categorical and a censored Gaussian data set, instead of dichotomizing the data into case-control observations, increases the accuracy of genomic breeding values predicted by Bayesian multilocus association models or by Bayesian G-BLUP. The amount of extra information of an ordinal data depends on the number of the classes and the distribution of the observations into the classes, higher number and more even distribution corresponding to higher information content. With a mildly to moderately (20–50% of observations) censored Gaussian data, the increase of the accuracy is substantial compared to binary data, but even if 80% of the observations are censored the remaining observations seem to possess some extra information.

Our results indicate that only a minor benefit is gained by using the correct threshold model compared with using the linear Gaussian model directly with a binary or ordinal data. These results are in concordance with the observations by Wang *et al.* (2013) under BayesB (Meuwissen *et al.* 2001) and BayesC*π* (Habier *et al.* 2011), and with the early observations of Meijering and Gianola (1985) for BLUP. However, for the same data set, under BayesA Wang *et al.* (2013) noted a substantial increase in the accuracy when the threshold model was used. Also, Meijering and Gianola (1985) observed that the threshold model was more reliable than BLUP if the number of fixed effects required in the mixed model was high. Even though our results do not confirm the practical superiority of the correct threshold model over the linear Gaussian model, we urge caution when applying a Gaussian model directly for an ordinal data. Some data sets may be less well-behaving than the ones we have studied and, as proven by Wang *et al.* (2013), different linear models may be less robust to the incompatible data.

The example analyses support the observation of Sorensen *et al.* (1998) that in the case of a censored Gaussian data the threshold model behaves better than the linear Gaussian model used directly. The benefit of the correct threshold model increases as the proportion of the censored observations increase. With 20% censored observations, the threshold model is slightly more accurate, whereas with 50% censored observations the accuracy gain is substantial. On the basis of our results, a heavily censored (80%) Gaussian data should not be analyzed with a linear Gaussian model, or, if analyzed, it should be dichotomized into a binary case-control data.

The sure independence screening (Fan and Lv 2008) works very well for such a strikingly simple method. It works so well probably because all it needs to do is to let all of the important markers pass to the next step whereas, because the final variable regularization is performed by the multilocus association model, it does not matter if unimportant ones are also selected. The optimal number of SNPs selected into the multilocus association model is data specific as it depends on the number of individuals in the learning set, and probably also on the genetic architecture of the trait and the LD structure. Additionally, multilocus association models with different shrinkage or variable selection mechanisms may be able to cope with different amount of oversaturation. The number of markers selected to the multilocus association model can be tuned into an optimal value similarly to the prior parameters. The model performance seems to be reasonably robust to the number of markers: in the pig data 10,000–20,000 markers produced almost identical accuracies with all of the response types. However, even though the sure independence screening seems to be a decent method indeed, and we have contented ourselves with using it for the marker preselection for the time being, there probably is room for improvement in this respect.

The difference in computation time between MCMC and (G)EM-algorithms is massive. Some authors have compared the speed difference between an MCMC and a MAP-estimation algorithm, for example, the fast BayesB implementation of Meuwissen *et al.* (2009) took 2–5 min to converge, whereas the MCMC-based BayesB required 47 hr. Using the same QTL-MAS data we have used, Shepherd *et al.* (2010) reported a computer time of few minutes for a MAP-estimation algorithm and 2 d for an MCMC algorithm (with a 2-GHz computer). Accordingly, with the same QTL-MAS data, the frequentist LASSO-LARS implementation of Usai *et al.* (2009) took more than 7 hr to converge. The enormity of the time difference can be illustrated by extrapolating the computer times reported by Shepherd *et al.* (2010) into the analysis of our 100 replicated QTL-MAS data sets: although the 100 analyses take 20 min with our GEM algorithm, with an MCMC algorithm the computation time would be stunning 600 hr, or 25 d. To get the best possible results, it seldom is sufficient to run an algorithm once, for instance, due to the tuning of the prior parameters and sensitivity analysis. The extremely short time requirement facilitates the adjusting for optimal performance, not to mention the usage of computer intensive techniques such as cross-validation and empirical threshold determination by phenotype permutation. Fast estimation is also extremely useful in simulation studies of entire breeding programs (see, *e.g.*, Pedersen *et al.* 2009; Axelsson *et al.* 2013).

The Bayesian threshold approach for binary, ordinal, and censored data enables the usage of a variety of different linear Gaussian model types—here we have demonstrated the method with Bayesian LASSO multilocus association model and Bayesian G-BLUP; however, depending on the genetic architecture and LD structure of the data, other variable selection or regularization methods than LASSO may be preferred. Whether the additional threshold layer actually increases the accuracy of the genomic breeding value estimates is questionable with a binary or an ordinal data but less so with a censored Gaussian data. To our experience it seems that especially with a heavily censored Gaussian data the threshold model should be used, but there is no harm in using it also with the binary and the ordinal phenotypes.

## Supplementary Material

Supporting Information

## Appendix A1

### Derivations of the fully conditional posterior densities

The joint posterior distribution of the unknown parameters, given the data, is proportional to the product of the joint prior and the likelihood. As the model parameters are considered *a priori* conditionally independent, the joint posterior density of the parameter vector under the multilocus association model (1) becomes

(A1)

The fully conditional posteriors of the parameters can be easily determined by selecting the terms including the parameter in question from the joint posterior. (For simplicity: * = ”the data, and the parameters except the one in question”)

As the prior density for the population intercept *β*_0_ is proportional to one, the fully conditional posterior distribution is proportional to the likelihood,

$$p\left(\beta_{0}|⋆\right)\propto \prod_{i=1}^{n}\text{exp}\left(-\frac{1}{2\sigma_{0}^{2}}{\left(y_{i}-\beta_{0}-\sum_{j=1}^{p}\beta_{j}x_{ij}\right)}^{2}\right).$$ (A2)

Note that we select from the joint posterior (A1) only the terms including *β*_0_. The right hand side of (A2) is a product of *n* kernels of normal probability density functions, with a common variance $\sigma_{0}^{2}$, and means $\left(y_{i}-\sum_{j=1}^{p}\beta_{j}x_{ij}\right)$, *i* = 1, …, *n*. The set of Gaussian functions is closed under multiplication, *i.e.* the product of normal densities is also a normal density, with the mean and the variance of the product density given by

$$\mu =\frac{\sum \frac{\mu_{i}}{\sigma_{i}^{2}}}{\sum \frac{1}{\sigma_{i}^{2}}}\;\text{and}\;\sigma^{2}=\frac{1}{\sum \frac{1}{\sigma_{i}^{2}}}$$

respectively. Hence, since in this case variance is same for all of the factors, the product density reduces to the sum of the means of the individual distributions, divided by *n*, while the variance of the product density is given simply by the variance of the individual distributions divided by *n*, leading to the fully conditional posterior density function given in (13).

For the fully conditional posterior distribution of the regression a coefficient *β_j_* we select from (A1) the terms including *β_j_*, located at the likelihood and at the prior $p\left(\beta_{j}|\sigma_{j}^{2}\right)$, and get

$$p\left(\beta_{j}|⋆\right)\propto \prod_{i=1}^{n}\text{exp}\left(-\frac{1}{2\sigma_{0}^{2}}{\left(y_{i}-\beta_{0}-\sum_{j=1}^{p}\beta_{j}x_{ij}\right)}^{2}\right)\text{exp}\left(-\frac{\beta_{j}^{2}}{2\sigma_{j}^{2}}\right).$$ (A3)

Here we have a product of two types of kernels of normal densities. The latter part of (A3) comes from the prior, it has mean 0 and variance $\sigma_{j}^{2}$. The former part represents the likelihood—regarding *β_j_* there are *n* kernels of normal distributions with means

$$\frac{1}{x_{ij}}\left(y_{i}-\beta_{0}-\sum_{l\neq j}\beta_{l}x_{il}\right),i=1,\ldots ,n,$$

and variances $\sigma_{0}^{2}/x_{ij}^{2}$, *i* = 1, …, *n*. As a product we get a normal distribution with a mean

$$\sum_{i=1}^{n}\left(\frac{x_{ij}}{\sigma_{0}^{2}}\left(y_{i}-\beta_{0}-\sum_{l\neq j}\beta_{l}x_{il}\right)\right)/\left(\sum_{i=1}^{n}\frac{x_{ij}^{2}}{\sigma_{0}^{2}}+\frac{1}{\sigma_{j}^{2}}\right)$$

and variance

$${\left(\sum_{i=1}^{n}\frac{x_{ij}^{2}}{\sigma_{0}^{2}}+\frac{1}{\sigma_{j}^{2}}\right)}^{-1},$$

which corresponds to the function in (10) when expanded by $\sigma_{0}^{2}$.

The residual variance $\sigma_{0}^{2}$ appears in the joint posterior density (A1) in the likelihood and in the prior density $p\left(\sigma_{0}^{2}\right)\propto 1/\sigma_{0}^{2}$, leading to

$$p\left(\sigma_{0}^{2}|⋆\right)\propto {\left(\sigma_{0}^{2}\right)}^{-\left(1+\frac{n}{2}\right)}\text{exp}\left(-\frac{1}{2\sigma_{0}^{2}}\sum_{i=1}^{n}{\left(y_{i}-\beta_{0}-\sum_{j=1}^{p}\beta_{j}x_{ij}\right)}^{2}\right).$$ (A4)

Regarding $\sigma_{0}^{2}$, this is an unnormalized probability density function of an inverse *χ*^2^-distribution (14), with *n* degrees of freedom and scale parameter equal to

$$\frac{1}{n}\sum_{i=1}^{n}{\left(y_{i}-\beta_{0}-\sum_{j=1}^{p}\beta_{j}x_{ij}\right)}^{2}.$$

The fully conditional posterior density for the variance of the marker effects $\sigma_{j}^{2}$ (11) is proportional to the product of the conditional prior for the marker effect $p\left(\beta_{j}|\sigma_{j}^{2}\right)$ and the exponential prior of the effect variance $p\left(\sigma_{j}^{2}|\lambda^{2}\right)$,

$$p\left(\sigma_{j}^{2}|⋆\right)\propto {\left(\sigma_{j}^{2}\right)}^{-1/2}\text{exp}\left(-\frac{\beta_{j}^{2}}{2\sigma_{j}^{2}}\right)\;\text{exp}\left(-\frac{\lambda^{2}\sigma_{j}^{2}}{2}\right),$$

for all *j* = 1, …, *p*. Since exponential density is not conjugate to normal density, we need to consider the inverse of the marker variance

$$p\left(\frac{1}{\sigma_{j}^{2}}|⋆\right)\propto {\left(\sigma_{j}^{2}\right)}^{1/2}\text{exp}\left(-\frac{\beta_{j}^{2}\sigma_{j}^{2}}{2}-\frac{\lambda^{2}}{2\sigma_{j}^{2}}\right){\left(\sigma_{j}^{2}\right)}^{-2},$$

where the last term is the Jacobian of the transformation $\sigma_{j}^{2}\to \sigma_{j}^{-2}$. By rearranging the terms, we get

$$p\left(\frac{1}{\sigma_{j}^{2}}|⋆\right)\propto {\left(\sigma_{j}^{2}\right)}^{-3/2}\text{exp}\left(-\frac{\lambda^{2}\left(\frac{\beta_{j}^{2}{\left(\sigma_{j}^{2}\right)}^{2}}{\lambda^{2}}+1\right)}{2\sigma_{j}^{2}}\right)$$

and completing the numerator of the exponent into square

$$p\left(\frac{1}{\sigma_{j}^{2}}|⋆\right)\propto {\left(\sigma_{j}^{2}\right)}^{-3/2}\text{exp}\left(-\frac{\lambda^{2}\left(\frac{\beta_{j}^{2}{\left(\sigma_{j}^{2}\right)}^{2}}{\lambda^{2}}-2\sigma_{j}^{2}\sqrt{\frac{\beta_{j}^{2}}{\lambda^{2}}}+1\right)}{2\sigma_{j}^{2}}-\frac{\lambda^{2}2\sigma_{j}^{2}\sqrt{\frac{\beta_{j}^{2}}{\lambda^{2}}}}{2\sigma_{j}^{2}}\right).$$

As $\sigma_{j}^{2}$ is canceled out from the last term inside the exponent, the term becomes constant and is left out. After that the exponent is expanded by $\lambda^{2}/\beta_{j}^{2}$ and we get

$$\begin{array}{c} p\left(\frac{1}{\sigma_{j}^{2}}|⋆\right)\propto {\left(\sigma_{j}^{2}\right)}^{-3/2}\text{exp}\left(-\frac{\lambda^{2}\left({\left(\sigma_{j}^{2}\right)}^{2}-2\sigma_{j}^{2}\sqrt{\frac{\lambda^{2}}{\beta_{j}^{2}}}+\frac{\lambda^{2}}{\beta_{j}^{2}}\right)}{2\sigma_{j}^{2}\frac{\lambda^{2}}{\beta_{j}^{2}}}\right)\\ ={\left(\sigma_{j}^{2}\right)}^{-3/2}\text{exp}\left(-\frac{\lambda^{2}{\left(\sigma_{j}^{2}-\sqrt{\frac{\lambda^{2}}{\beta_{j}^{2}}}\right)}^{2}}{2\sigma_{j}^{2}\frac{\lambda^{2}}{\beta_{j}^{2}}}\right),j=1,\ldots ,p. \end{array}$$ (A5)

This is an inverse-Gaussian probability density function (Chhikara and Folks 1989) with mean *μ*′ and shape *λ*′

$$\mu \prime =\sqrt{\frac{\lambda^{2}}{\beta_{j}^{2}}}\;\text{and }\lambda \prime =\lambda^{2},$$

the parametrization of the inverse-Gaussian density being

$$f\left(x|\mu \prime ,\lambda \prime \right)\propto x^{-3/2}\text{exp}\left(-\frac{\lambda \prime {\left(x-\mu \right)}^{2}}{2{\left(\mu \prime \right)}^{2}x}\right).$$

The LASSO parameter *λ*^2^ occurs in the joint posterior (A1) in the exponential priors for the effect variances $\sigma_{j}^{2}$ and in the gamma prior for the LASSO parameter.

$$\begin{array}{c} p\left(\lambda^{2}|⋆\right)\propto \prod_{j=1}^{p}\left(\frac{\lambda^{2}}{2}\text{exp}\left(-\frac{\lambda^{2}\sigma_{j}^{2}}{2}\right)\right){\left(\lambda^{2}\right)}^{\kappa -1}\text{exp}\left(-\xi \lambda^{2}\right)\\ \propto {\left(\lambda^{2}\right)}^{\kappa +p-1}\text{exp}\left(-\lambda^{2}\left(\xi +\sum_{j=1}^{p}\frac{\sigma_{j}^{2}}{2}\right)\right), \end{array}$$ (A6)

that is an unnormalized gamma probability density function with shape *κ* + *p* and rate

$$\xi +\sum_{j=1}^{p}\frac{\sigma_{j}^{2}}{2},$$

corresponding to (12).

## Appendix A2

### The GEM algorithms

Below we present the GEM algorithms for *maximum a posteriori* parameter estimation under the threshold model combined with the Bayesian LASSO and the Bayesian G-BLUP. In both algorithms, the steps are repeated until convergence. Matlab codes of the algorithms are included as Supporting Information, File S1.

### GEM algorithm for the multilocus association model

1. Set initial values for parameter vectors. We use zeros for *β*_0_, **β**, and the latent variable **y**, and small positive values, namely 0.1, for the variances **σ**^2^ and $\sigma_{0}^{2}$ (when estimated), and for the LASSO parameter *λ*^2^. The initial value for the threshold vector $t^{⋆}=\left(\frac{1}{K-2},\frac{2}{K-2},\ldots ,\frac{K-2}{K-2}\right)$.
2. Update the values of the latent variable **y** by replacing the current values *y_i_* with the expected values of the truncated normal distribution (6),
  $$y_{i}:=\mu_{i}+\frac{\phi \left(t_{k-1}-\mu_{i}\right)-\phi \left(t_{k}-\mu_{i}\right)}{\Phi \left(t_{k}-\mu_{i}\right)-\Phi \left(t_{k-1}-\mu_{i}\right)},$$
  where $\mu_{i}=\beta_{0}+\sum_{j=1}^{p}\beta_{j}x_{ij}$, and *ϕ*(⋅) and Φ(⋅) denote the standard normal density function and distribution function, respectively. If the actual Gaussian phenotype **y** is fully available, this step naturally becomes obsolete.
3. Update the K − 2 unknown thresholds to their conditional expectations,
  $$t_{k}:=\frac{1}{2}\left(\text{max}\left(y_{i}|w_{i}=k\right)+\text{min}\left(y_{i}|w_{i}=k+1\right)\right)$$
  for all *k* = 2, …, K-1. This step is bypassed if i) the phenotype is Gaussian and fully observed, ii) the phenotype is binary, *i.e. K* = 2, or iii) the phenotype is left or right censored (no unknown thresholds).
4. Maximize the posterior distributions of *β*_0_ and *β_j_* (for all j) by substituting the fully conditional expectations for the current values of the parameters, one at the time.
  $$\begin{array}{l} \beta_{0}:=\frac{1}{n}\sum_{i=1}^{n}\left(y_{i}-\sum_{j=1}^{p}\beta_{j}x_{ij}\right)\text{and}\\ \beta_{j}:=\sum_{i=1}^{n}x_{ij}\left(y_{i}-\beta_{0}-\sum_{l\neq j}\beta_{l}x_{il}\right)/\left(\sum_{i=1}^{n}{\left(x_{ij}\right)}^{2}+\frac{\sigma_{0}^{2}}{\sigma_{j}^{2}}\right). \end{array}$$
  Note that the residual variance $\sigma_{0}^{2}=1$ unless the Gaussian phenotype is fully observed.
5. In case of a fully observed Gaussian phenotype, update the error variance $\sigma_{0}^{2}$ into its conditional expectation
  $$\sigma_{0}^{2}:=\frac{1}{n-2}\sum_{i=1}^{n}{\left(y_{i}-\beta_{0}-\sum_{j=1}^{p}\beta_{j}x_{ij}\right)}^{2},\;\text{for}n>2.$$
6. Update the effect variances $\sigma_{j}^{2}$ (for all j) to their conditional expectations. The precision, or inverse of the variance parameter $\sigma_{j}^{2}$, has an inverse-Gaussian fully conditional posterior distribution leading to following fully conditional expectation for the effect variances
  $$\sigma_{j}^{2}:=\frac{\left|\beta_{j}\right|}{\lambda }.$$
7. Update the hyperparameter λ^2^ into its conditional expectation
  $$\lambda^{2}:=\left(1+p\right){\left(\xi +\sum_{j=1}^{p}\frac{\sigma_{j}^{2}}{2}\right)}^{-1}\;\text{for prior shape }\kappa =1.$$

### GEM algorithm for the Bayesian G-BLUP

1. Set initial values for parameter vectors; zeros for *β*_0_, **u** and **y**, and 0.1 for $\sigma_{u}^{2}$ and $\sigma_{0}^{2}$ (when estimated), $t^{⋆}=\left(\frac{1}{K-2},\frac{2}{K-2},\ldots ,\frac{K-2}{K-2}\right)$.
2. Update the latent variable **y** as previously, except that here *μ*_i_ = *β*_0_ + *u_i_*.
3. Update the thresholds as previously.
4. Update the population intercept *β*_0_ into its fully conditional expectation
  $$\beta_{0}:=\frac{1}{n}\sum_{i=1}^{n}\left(y_{i}-u_{i}\right).$$
5. Update the polygenic effects **u** by replacing the current values with the conditional expectations
  $$u:={\left({\mathrm{Z\prime}}^{\prime }Z+\frac{\sigma_{0}^{2}}{\sigma_{u}^{2}}G^{-1}\right)}^{-1}{\mathrm{Z\prime}}^{\prime }\left(y-\beta_{0}\right),$$
  where **G**^−1^ denotes a generalized inverse of the realized relationship matrix. Note that the residual variance $\sigma_{0}^{2}=1$ except in the fully observed Gaussian phenotype case.
6. In case of a fully observed Gaussian phenotype, update the error variance $\sigma_{0}^{2}$ into its fully conditional expectation
  $$\sigma_{0}^{2}:=\frac{1}{n-2}\left(y-\beta_{0}-Zu\right)\prime \left(y-\beta_{0}-Zu\right).$$
7. Replace the additive genetic variance component $\sigma_{u}^{2}$ with its conditional expectation
  $$\sigma_{u}^{2}:=\frac{1}{N}\left({\mathrm{u\prime}}^{\prime }G^{-1}u+2\tau^{2}\right)\;\text{for}\;\text{prior}\;\text{shape }\nu =2,$$
  where and *N* is the total number of individuals in the learning and validation sets (*i.e.* the dimension of **G** is *N* × *N*), and **G**^−1^ is a generalized inverse of the realized relationship matrix.
