# Supplementary material for: Fast Genomic Predictions via Bayesian G-BLUP and Multilocus Models of Threshold Traits Including Censored Gaussian Data
Source: G3 (Bethesda). 2013 Sep 1;3(9):1511–23. doi: 10.1534/g3.113.007096 (PMC3755911; doi:10.1534/g3.113.007096)
Supplement: Supporting Information [file supp_3_9_1511__index.html]

Fast Genomic Predictions via Bayesian G-BLUP and Multilocus Models of Threshold Traits Including Censored Gaussian Data — Supporting Information 

# Fast Genomic Predictions via Bayesian G-BLUP and Multilocus Models of Threshold Traits Including Censored Gaussian Data

## Supporting Information for Kärkkäinen and Sillanpää, 2013

**Files in this Data Supplement:**

- File S1 - MATLAB codes of GEM-algorithms (.zip, 3 KB)
